# The poorly understood yet potent risk of pulmonary artery thrombosis in-situ in Post-Acute COVID-19 syndrome

**DOI:** 10.1186/s13019-023-02147-y

**Published:** 2023-01-20

**Authors:** Alvin Oliver Payus, Constance Liew Sat Lin, Azliza Ibrahim

**Affiliations:** 1 Medicine Department, Faculty of Medicine and Health Science, Universiti Malaysia Sabah (UMS), Jalan UMS, 88400 Kota Kinabalu, Sabah Malaysia; 2 Department of Neurology, Hospital Pengajar Universiti Putra Malaysia, Persiaran Mardi - UPM, 43400 Serdang, Selangor Malaysia

## Abstract

Pulmonary artery thrombosis in-situ is a term used to describe a pulmonary embolism occurs in the absence of deep vein thrombosis in the lower extremities. Most cases occur in a patient who had a recent traumatic injury to the chest. Other risk factors include the presence of hypercoagulable conditions, including inflammatory state, hypoxia and vascular endothelial injury. Although it has been discussed extensively in the acute COVID-19 disease, pulmonary artery thrombosis in-situ that occur in the setting of Post-Acute COVID-19 syndrome is not commonly reported and poorly understood.

We read with great interest the article by Alrifae GM et al., entitled “Post-Acute COVID-19 syndrome (PACS) right atrioventricular and vena cava thrombus on top of a myxoma. A Case report " which was recently published in the volume 17, Article: 261 (2022) [1]. Firstly, we would like to praise the authors for their effort in describing a case of coronavirus disease 2019 (COVID-19)-related intracardiac thrombosis encasing a cardiac myxoma in the setting of post-acute phase of COVID-19 syndrome. The authors have nicely written the case in great detail and conclude the occurrence of intracardiac thrombosis in atrial myxoma as a sequalae of COVID-19 which occurs four months prior this presentation. We strongly support the statement by the authors that thrombosis can developed in situ, especially in the background of hyperinflammatory condition, like COVID-19 which leads to hypercoagulable state [2, 3]. However, there is some important information that is missing in the article, which is the background history of the patient during her COVID-19 illness especially her inflammatory markers, and also the list of regular medication that she is taking. We believe this information is vital to determine the aetiology and prognosis of the condition. Apart from that, we believe a follow-up reporting is warranted on the rate and status of recovery after rehabilitation and the presence of other PACS symptoms. Nevertheless, we agree with the authors that intracardiac thrombosis that occur over cardiac myxomas as a sequalae of PACS have not been well described in the literature. Therefore, we believe this study will be a potential catalyst to trigger future studies to improve the treatment and prevention of thrombosis and other cardiovascular complications associated with COVID-19.

## Data Availability

Not applicable.
